# Use of a Novel Natural Language Processing Utility to Extract Structured Data From Free-Text Medical Notes

**DOI:** 10.7759/cureus.96838

**Published:** 2025-11-14

**Authors:** John Culhane

**Affiliations:** 1 Department of Surgery, Saint Louis University, St. Louis, USA

**Keywords:** computer-aided diagnosis, free text notes, intensive care unit, natural language processing (nlp), research methodology

## Abstract

Introduction

Electronic medical records (EMRs) contain a large amount of clinical data potentially useful for research. A barrier to the use of this data is that much of it is unstructured, mainly in the form of free-text clinical notes. Software utilities can help with extraction and conversion of this information into a usable form. Traditionally, this was performed with rule-based natural language processing (NLP) software, but, recently, large language models based on machine learning have been applied to this task.

Methods

A set of software utilities called Note Language Processing (NoteLP) were developed to extract information from free-text notes and convert it to datasets in standard format. A manual data search was performed in the Epic EMR to serve as the reference standard for the time required to conduct a search manually. Twenty-two sample research projects were designed. The time required to extract the required data using NoteLP was recorded and compared to the time that would be required per patient to retrieve the same data via manual search. Eleven of the search criteria for our sample projects that had corresponding ICD-9 codes were selected. A gold standard for accuracy was established via manual review. Sensitivity and specificity of ICD-9 and note extracted data were calculated versus the gold standard.

Results

The reference standard was established at 63.4 seconds per patient to manually classify one condition per patient via a manual Epic search. NoteLP required a mean of 37.5 seconds per patient to retrieve data for the sample projects involving ICD-9 comparisons. A further set of more extensive searches required a mean of 16.6 seconds per patient (p<0.001 for all time comparisons of NoteLP versus manual search). The mean ICD-9 sensitivity was 0.65 versus the mean note sensitivity of 0.98 (p<0.001). The mean ICD-9 specificity was 0.93 versus the mean note specificity of 0.94 (p=0.65).

Conclusion

The use of NoteLP is more efficient than a manual search. Its accuracy compares favorably with manual coding, achieving greater sensitivity and equal specificity. Rule-based NLP utilities such as NoteLP remain a valuable tool for the extraction of research information from unstructured medical text.

## Introduction

This study aims to compare the speed and accuracy of a new text-extraction software package called NoteLP. The speed of text extraction is compared to manual chart review, and the accuracy is compared to the International Classification of Disease (ICD-9) coding. The hypothesis is that the system is faster than manual chart review and at least as accurate as coding performed by human registrars. Modern electronic medical records (EMRs) have created vast quantities of medical data stored in digital format [1]. The extraction of this data in a form useful for research is known as data mining. Information obtained from data mining has tremendous potential value for clinical investigation; however, a barrier to the use of this data is that much of it is unstructured, mainly in the form of free-text clinical notes [2].

The traditional method to retrieve information from free text is the manual abstraction of data into standardized categories such as the International Classification of Disease (ICD) and current procedural terminology (CPT) codes. These codes can then be incorporated into the EMR database or used to create registries, both local and public. A disadvantage of this strategy is the reliance on manual chart review by human registrars. Manual abstraction is time-consuming and expensive. It is often designed for billing rather than research, which results in codes based on financial rather than scientific criteria. Thus, pre-defined categories may not be available for variables which are mainly of scientific interest.

An alternative to direct chart review is automated or semi-automated review with the aid of artificial intelligence. Rule-based natural language processing (NLP) has been used for decades to identify entities in free text and determine their meaning in context [3]. A drawback of rule-based systems is that a great deal of human input is required to derive and apply the rules used to interpret the meaning of text strings. Additionally, unique characteristics of clinical notes such as jargon, abbreviations, and medical slang limit the use of standardized NLP libraries [4].

Recently, systems based on machine learning (ML) have superseded rule-based NLP for many applications, most notably with the advent of generative large language models (LLMs) such as ChatGPT [5]. While LLMs are certainly labor-saving, they suffer some disadvantages when used to generate datasets for research. Models based on statistics and neural networks rely on layers of processing behind the scenes. This results in a black box that prevents researchers from investigating how a given result was derived. LLMs are based on the statistical likelihood of associated patterns in text independent of the underlying meaning. This makes LLMs vulnerable to hallucinations in which false or even fictitious results are generated [6].

Therefore, despite the recent success of LLMs, for reasons of accuracy and transparency, rule-based systems may still have a valuable role to play in medical research. To help define the role of specialized NLP in the era of LLMs, new software was developed by the author to extract information from free-text notes, classify it according to meaning, and convert it to structured datasets. This system is an example of supervised rule-based AI requiring human input to create and maintain classification logic. This logic can then be generalized to other searches, allowing greater future autonomy. As with other text extraction utilities, this system is designed to improve efficiency, but an even greater focus is placed on transparency and ease of reviewing and auditing results.

## Materials and methods

Data source

This is a retrospective registry review that measures the speed and accuracy of extracting information from unstructured EMR data via NLP techniques versus manual chart review. The data source is the Medical Information Mart for Intensive Care (MIMIC) III database. MIMIC is a public registry of intensive care unit (ICU) data from Beth Israel Deaconess Hospital in Boston [7]. While not the most recent version of this database, MIMIC III was chosen because it includes all daily progress notes. Patients are selected from a mixed medical and surgical population that includes trauma. For this project, 890,000 notes from MIMIC were selected as clinically relevant. These are mainly history and physical examinations, daily progress notes, and discharge summaries.

Inclusion criteria

Inclusion criteria were all patients with only a single admission documented in MIMIC. Patients with multiple admissions were excluded because the classification system includes the distinction between acute versus chronic conditions. Acuity generally depends on whether the condition was discovered during a given index admission. This classification was inconsistent for temporally distinct yet closely spaced admissions; therefore, to simplify the analysis, only single-admission patients were included. For rare conditions, all single-admission patients were included. For more common conditions, for logistical reasons, a subset of a manageable number was chosen. The admissions extended over 11 years from 2001 through 2012. Ages 17 to 90+ were included. MIMIC groups together all ages greater than 90 as part of their de-identification algorithm. The exclusion criteria also omit age under 17. All patients in the data set meeting the above criteria were included in the study. Sample size was determined by the number of patients who met the inclusion criteria.

Software function

Note Language Processing (NoteLP) is a suite of free-text analysis software, coded in Python at our institution, which extracts information from medical notes and converts the information to structured data sets. These data sets can then be modified or analyzed with standard spreadsheet and statistical software. For greater accessibility, the system is based on the readily available software packages of the open-source Python Integrated Development and Learning Environment (IDLE) and Microsoft Notepad, although any text editor and implementation of Python can be used. The computer used was a standard desktop personal computer with an Intel Core i7 processor.

To prepare the system for use, the full text of the selected clinical notes from the EMR is downloaded into a database. The resulting note file consists of plain text in a comma-separated value (CSV) format. The notes are preprocessed for use in all searches. Stop words (words less essential to meaning such as “the”) are removed, letters are converted to lower case, and punctuation and other symbols are standardized.

The data extraction for each search takes place in four steps:

Step one begins with identifying the pattern of interest, often a disease or condition. The initial exploration utility then locates and organizes all instances of the search pattern in the EMR clinical notes. The retrieved search patterns and surrounding text comprise syntactic groups that are used to create an intermediate database. This is composed of plain text and is fully searchable. The search pattern and extracted syntactic groups are processed using the standard NLP techniques of tokenizing, stemming, and lemmatization [3]. Simple examples are isolating the roots “delir” for delirium and delirious and “decub” for decubitus ulcer. Terms with similar meaning are grouped together, for instance, pneumonia with its common abbreviation pna. This initial step of parsing the 890,000 notes takes an average of about 60 seconds. Subsequent searches are nearly instantaneous because the subset of relevant notes is extracted and used for future analysis.

The second step is to categorize each instance of the pattern according to its meaning in context. This is done by querying a custom library of standard and specialized medical phrases and linguistic conventions. The library is easily modified and expands with each search. Depending on the needs of the project, the user can custom-define any category, but the following categories were most useful for retrospective chart review study designs:

1. The condition is acutely present during the index admission. Typical surrounding words are “currently with”, “admitted with”, and “chief complaint”.

2. The patient has a history of the condition, or the condition is chronic and not relevant to ongoing treatment. The search pattern is commonly associated with words such as “history of”, “previous hospitalization for”, and “as a child”.

3. The condition is specified as not present. Typically associated words are “no evidence of”, “negative for”, “doubt”, and “without”.

4. The patient is being evaluated for the condition as indicated by phrases such as “may have”, “suspected”, and “assessment for”.

5. The search term has a completely different meaning in the context of the note, for instance, decubitus ulcer versus decubitus xray, joint aspiration versus aspiration pneumonia, or lupus anticoagulant versus lupus erythematosus.

The program automatically categorizes all patterns found in the library. This represents the application of supervised artificial intelligence. All instances not found in the library are listed as uncategorized and must be classified manually. The software provides a utility for helping the user efficiently assign meaning to the uncategorized instances. It lists the unassigned instances in a form convenient for editing with a specified number of words before and after the search pattern.

The user starts by reviewing short word groups and then increases the length as necessary until all have been displayed with sufficient context to specify the meaning. Results are applied to the entire list as the process continues, shrinking the list and making the task more efficient. This generates a project-specific library of word groups with assigned meanings. Those of broader applicability are placed in the general library for use in future projects. This is the most time-consuming part of the process, but it is still faster than a manual chart review. 

The third step is an optional manual review or audit of the results of the search and categorization. The program generates specialized reports that can be sampled or reviewed in their entirety. This allows the user to balance precision and efficiency for a given project.

The fourth step is conversion to a dataset in standard format. The program automatically creates a CSV file with cases consisting of a key, typically based on the patient’s medical record number, and a series of binary variables representing conditions flagged as present or absent. Instances of search patterns identified as negative, out of specified temporal context, or ambiguous are typically classified as absent. These files can be combined with each other or with registry data using any statistical software package or spreadsheet.

Measure of efficiency

A manual chart review was performed to generate a reference standard for the time required to retrieve data via the standard Epic user interface. The project chosen was a straightforward chart review with standard terminology and simple inclusion criteria. The task was to identify colonization or infection with methicillin-resistant *Staphylococcus aureus* (MRSA) for 100 sequential trauma patients from the institutional registry. This manual search allowed measurement of the fixed overhead required to retrieve each record and the time necessary to manually extract the variable of interest using the built-in Epic text search utility. Time required to retrieve the patient's record, search for the pattern of interest, and record the data in a spreadsheet was all combined into the time per patient. The next step was to design 22 simulated research projects that require identification of a condition (phenotyping). NoteLP was used to extract the relevant information from MIMIC free-text notes, including retrieval and assignment of contextual meaning. The time required per patient was recorded.

Measure of accuracy

Eleven of the 22 simulated research projects were selected. These involve identifying conditions with specific corresponding ICD-9 codes included in MIMIC. A gold standard was established by manual review of structured and unstructured data from the MIMIC registry. When considering the gold standard, it should be noted that NoteLP is designed as a research tool. The gold standard defined for each project is the research definition, based on the inclusion criteria for the design of each simulated study. This does not mean that discrepant ICD-9 codes are wrong but that they categorize patients incorrectly in the context of a given study design. Three reviewers, blinded to NoteLP results, participated in the manual review. Disagreements were resolved by an open discussion modeled after a review of data results typical of an actual research project.

Accuracy of results obtained by note extraction versus the ICD-9 codes was then compared. Sensitivity and specificity were calculated for the ICD-9 codes and software results versus the gold standard. Confidence intervals were generated for the sensitivity and specificity. The significance threshold is defined as non-overlapping 95% confidence intervals. One type of potential classification error came up frequently during analysis: ICD-9 false negative due to omitting a chronic condition. This type of error was tracked separately and displayed qualitatively.

Coding was done with Python version 3.12.0. Statistics were done with Python using the sklearn confusion matrix module and scipy.stats modules: sem for standard error of the mean, ttest_ind for t-test, and t.ppf to generate confidence intervals.

The NoteLP code is open-source and can be accessed in a Github repository. The public repository is called NoteLP (Github.com/jculhane1/NoteLP).

## Results

Time for standardized manual search

A mean of 63.4 seconds (95% confidence interval: 58.2 to 68.6) was required per patient via a manual Epic search to determine whether they had ever been colonized or infected with MRSA. This includes 32 seconds per patient needed to retrieve the medical record with the Epic user interface.

Measurement of efficiency

Table 1 displays the time required per patient to perform a search for selected conditions within MIMIC free-text progress notes. In general, the time per patient diminishes with searches that yield more positive results.

**Table 1:** Time required to retrieve and classify instances of text strings matching search patterns for selected conditions. The mean data extraction time per patient for the ICD-9 comparison questions was 37.5 seconds and 16.6 seconds for the other searches. P-value was <0.001 for mean NoteLP search time versus the manual search reference standard. Significance testing was performed using t-test. The first 11 rows are the conditions used for the ICD-9 comparison, the next eight rows are conditions that tended to yield more positive results, selected to test the efficiency of the system, and the final three rows are events rather than conditions.

Condition	Patients Screened	Patients Positive for Text Pattern	Seconds Per Patient
Central cord syndrome	30,941	73	71
Lupus	10,000	138	59
Intestinal volvulus	30,941	146	54
Brain death	30,941	209	46
Meningitis	10,000	276	36
Esophageal varices	10,000	301	34
Pulmonary embolism	1,000	244	32
Aspergillus	30,941	264	26
Osteomyelitis	10,000	235	26
Diabetes insipidus	30,941	497	15
Endocarditis	10,000	417	14
Status post splenectomy	10,000	99	69
Intraperitoneal abscess	46,521	679	27
Portal vein thrombosis	46,521	244	13
Pneumonia	10,000	2,580	6
Delirium	10,000	477	6
Aspiration	10,000	1,455	5
Cocaine use	46,521	3,053	4
Decubitus ulcer	46,521	2,354	4
Pulling out gastrostomy	46,521	119	22
Code purple (agitation)	46,521	160	21
Kicking staff	46,521	1226	6

Measurement of accuracy

Table 2 reflects the accuracy of ICD-9 codes to identify conditions selected as the subject of potential research projects. This measures the performance of registrar generated billing codes applied for a scientific purpose.

**Table 2:** Sensitivity and specificity of ICD-9 codes versus the gold standard for selected conditions. Mean ICD-9 sensitivity is 0.65. Mean ICD-9 specificity is 0.93. Confidence intervals are 95%. In general, sensitivity for ICD-9 codes is much lower than specificity.

Condition	n	ICD-9 Sensitivity	ICD-9 Specificity
Aspergillosis	264	0.69 (0.57-0.78)	0.98 (0.95-0.99)
Lupus	138	0.80 (0.67-0.88)	0.99 (0.94-0.99)
Brain death	209	0.04 (0.01-0.09)	0.96 (0.90-0.98)
Meningitis	276	0.89 (0.79-0.95)	0.92 (0.87-0.95)
Diabetes insipidus	497	0.56 (0.45-0.66)	0.98 (0.96-0.99)
Pulmonary embolism	244	0.96 (0.82-0.99)	0.99 (0.97-0.99)
Intestinal volvulus	146	0.72 (0.58-0.82)	0.87 (0.79-0.92)
Esophageal varices	301	0.55 (0.48-0.62)	0.92 (0.85-0.96)
Central cord syndrome	73	0.59 (0.43-0.72)	0.94 (0.80-0.98)
Osteomyelitis	235	0.53 (0.44-0.62)	0.74 (0.66-0.81)
Endocarditis	417	0.85 (0.75-0.92)	0.97 (0.94-0.98)

Table 3 reflects the performance of the note extraction software for the same selected conditions. This search was performed to extract scientific data with specific research questions in mind.

**Table 3:** Sensitivity and specificity of NoteLP versus the gold standard for selected conditions. Mean note sensitivity is 0.98. Mean note specificity is 0.94. P<0.001 for the difference between mean ICD-9 sensitivity and mean note sensitivity. P=0.65 for the difference between mean ICD-9 specificity and mean note specificity. Significance testing was performed using t-test. Confidence intervals are 95%.

Condition	n	Note Sensitivity	Note Specificity
Aspergillosis	264	0.96 (0.88-0.98)	0.95 (0.91-0.97)
Lupus	138	1.0 (0.93-1.0)	0.98 (0.92-0.99)
Brain death	209	1.0 (0.97-1.0)	0.94 (0.87-0.97)
Meningitis	276	0.89 (0.79-0.95)	0.99 (0.96-0.99)
Diabetes insipidus	497	0.98 (0.92-0.99)	0.96 (0.94-0.98)
Pulmonary embolism	244	0.96 (0.82-0.99)	0.99 (0.97-0.99)
Intestinal volvulus	146	0.98 (0.90-0.99)	0.95 (0.88-0.98)
Esophageal varices	301	1.0 (0.98-1.0)	0.85 (0.77-0.91)
Central cord syndrome	73	1.0 (0.91-1.0)	0.91 (0.76-0.97)
Osteomyelitis	235	0.98 (0.92-0.99)	0.97 (0.92-0.99)
Endocarditis	417	1.0 (0.95-1.0)	0.90 (0.86-0.92)

Note sensitivity was significantly greater for Aspergillus, lupus, brain death, diabetes insipidus, intestinal volvulus, esophageal varices, central cord syndrome, osteomyelitis, and endocarditis. ICD-9 was not more sensitive for any condition. ICD-9 specificity was significantly greater for Aspergillus, diabetes insipidus, esophageal varices, and endocarditis. Note specificity was significantly greater for meningitis, intestinal volvulus, and osteomyelitis. Significance for all was at the 0.05 level based on non-overlapping 95% confidence intervals.

Table 4 displays the frequency of the omission of chronic conditions accounting for false negative ICD-9 coding.

**Table 4:** Chronic conditions not assigned ICD-9 codes for admissions reviewed in this study, resulting in false-negative categorization. Endocarditis shows by far the most false-negative ICD-9 assignment due to the chronic nature of a condition.

Condition	Chronic History
Aspergillosis	2
Diabetes insipidus	1
Intestinal volvulus	2
Central cord syndrome	2
Endocarditis	16

## Discussion

The near-universal adoption of EMRs has resulted in a vast trove of data potentially available for research [1]. While the electronic format makes patient information readily available to providers, efficient access and organization/classification remain a barrier to its use in clinical investigation. The user interface and data structure of an EMR are designed for clinical practice, making electronic health care data better suited to patient care and billing than research. A feature that is a particular challenge to researchers is the large proportion of information stored in an unstructured format. Around 80% of EMR data is unstructured, principally in the form of free-text notes [8]. Free text is a natural and flexible way for providers to communicate but does not lend itself to quantitative analysis without transformation.

A logical choice for abstracting unstructured computerized data is to use software to automate the process. Computer-assisted text analysis falls into two broad categories: rule-based systems, which depend on manually specified decision rules, and systems based on ML, which rely on statistical analysis and neural networks to recognize patterns. Text extraction by classic rule-based NLP involves identifying a condition of interest by matching search terms and analyzing their meaning in the context of adjacent syntactic patterns. This analysis relies on rules of grammar and syntax. Rule-based data extraction tends to yield superior results, but rules specific to a given dataset must be handcrafted, which is labor-intensive and makes the utility less generally applicable [9].

An additional barrier to the use of NLP techniques for medical text analysis is the difference between notes and commonly encountered speech patterns. Medical notes are a highly specialized jargon, a cross between formal narrative structure and a combination of lists, shorthand, raw data, and colloquial expressions. This creates unique linguistic properties that may vary between different types of clinical notes [4]. This also limits the utility of commonly available general purpose NLP software libraries.

One solution to the complex and time-consuming task of setting up elaborate rule systems is to allow the algorithm to learn from the data itself. This concept is embodied in ML, most recently in the form of LLMs. Modern LLMs are statistical or neural network based allowing autonomous processing of training data. This permits the system to derive its own rules, which it can then apply to novel prompts including unstructured data. This feature is certainly labor-saving and has achieved some impressive results in the medical field. Specialized medical LLMs have become so sophisticated that they can pass the USMLE [6]. Other medical applications are generating documents to communicate with patients, aiding diagnosis and clinical decision-making [10].

LLMs have been applied to medical research as well. They have been used to review literature and prepare manuscripts [10]. They have also been used to transform unstructured data. Chen et al. found that LLMs were labor-saving when used to gather oncologic data [11]. Huang et al. used ChatGPT to extract tumor characteristics from pathology reports with an average sensitivity of 0.89 and specificity of 0.89 [5].

The application of LLMs in medical research is limited by some of their inherent drawbacks. ML generates content using complex statistics with multiple layers. This process conceals the methods used to derive a given response. The result is a black box, inaccessible to audit [3,10]. This problem is further exacerbated in commercial systems whose proprietary software is unavailable for review due to copyright. LLMs cannot guarantee the veracity of their training data. Their results rely on the statistical likelihood of one word following another rather than the meaning of the underlying content. This may result in hallucinations where the output of the LLM contains falsehoods or even complete inventions. In contrast to fields such as marketing, false results are a particular problem in medicine where precision is important. Medical research may directly or indirectly influence decisions that affect patient safety. Yoo and Kim found that data analysis using LLM was fraught with many errors in coding [12]. In a review of data extraction aided by LLMs, Chen et al. found that nine (38%) of 24 reviewed studies reported problems with accuracy [11].

Despite the drawbacks of LLMs, their recent spectacular success in many fields has led some to question whether rule-based NLP for text analysis is obsolete [13]. The results of the current study show that it can still play a valuable role. NoteLP is more efficient than a manual chart search. Performance was about twice as fast for a small straightforward project, optimized for efficient manual retrieval with the Epic user interface. For larger and more complex searches, economy of scale becomes apparent with NoteLP automatically classifying more data, leaving less for manual review. NoteLP can perform longer searches about 5 times faster than a manual chart review (Table 1). 

Accuracy also compares favorably. NoteLP is consistently more sensitive than ICD-9 codes assigned manually by registrars. Indeed, NoteLP achieved perfect sensitivity for many note-derived variables (Tables 2, 3). This is likely related to the ever-greater verbosity of electronic clinical notes. EMRs make it easy to drop copied text and other results into text, making notes increasingly longer and more redundant. This trend is prompted at least in part due to medicolegal concerns [3]. The resulting phenomenon is known as note bloat [14]. While this may make finding the relevant part of a note more difficult in clinical practice, this greater inclusiveness may help data retrieval. Note length has increased to the extent that it is rare that a clinically relevant condition would never appear in any note including the history and physical and discharge summary [14].

Various investigators have capitalized on the inclusiveness of EMR notes by using specialized NLP software to extract information. Zeng et al. developed a utility to extract the principal diagnosis, comorbidity, and smoking status from free-text note with an accuracy of 0.82 to 0.87, and 0.90, respectively [15]. Penz et al. used a combination of a phrase matching algorithm and NLP software to identify complications of central venous catheter placement. They found a sensitivity of 72.0% and a specificity of 80.1% versus manual chart review [16]. Liao classified patients with rheumatoid arthritis using NLP and ICD-9 coding. The combined method achieved a PPV of 0.94 versus 0.88 for ICD codes alone [17]. Ford et al., in a systematic review of text extraction, found a sensitivity of 78% for diagnostic codes and text extraction versus 62% for codes only (p=0.25) [18].

The pattern of discrepancies between ICD-9 and note-derived results was also revealing. False-negative ICD-9 coding was often due to the omission of chronic conditions (Table 4). This may lead researchers to systematically underestimate the population prevalence of chronic versus acute conditions. Although personal history of the condition is an available ICD category, this was used inconsistently. Endocarditis was particularly vulnerable to this type of misclassification possibly due to complex diagnostic criteria and uncertain distinction between active disease and sequelae.

Future

The classification files generated by NoteLP can be used as training data for software with more autonomy. Work is underway to improve the user interface by creating a dedicated text editor with built-in note processing utilities.

Limitations

MIMIC is the most comprehensive public medical registry available, but it is possible that some records for a given patient could be omitted. A condition identified by ICD-9 but not mentioned in any note may be based on outside information or a note missing from the registry. The gold standard is inherently subjective and based partially on information contained in notes favoring accuracy of note-derived data. Due to its retrospective design, this study cannot establish causal superiority beyond the specific dataset used (MIMIC-III). The system requires a data set of all free-text notes for the patient population. This could represent a security concern for some institutions.

## Conclusions

NoteLP is a rule-based set of NLP utilities designed to extract information from free-text notes for research. The use of NoteLP for peer-reviewed articles and its performance versus ICD-9 coding in this validation study suggest that there is still a potential utility for rule-based algorithms in the age of LLMs. The strengths of NoteLP are simplicity, transparency, explainability, and ease of auditing results. NoteLP saves time versus manual data extraction via direct chart review, yet its greater efficiency does not sacrifice accuracy shown by its greater sensitivity and comparable specificity for research questions compared to ICD-9 coding performed by registrars. This study also suggests that medical literature may systematically underreport the prevalence of certain conditions, especially chronic, by relying on ICD codes to identify affected patients.

LLM technology is not standing still. Its performance will no doubt improve to the point where it can match or overtake human coders. However, even when a set of previously abstracted data is available or one is confident in the output of a generative LLM, there are advantages to using data derived directly from free text by NLP utilities. Researchers may want to maintain fine control over the data extraction process by reviewing and auditing the intermediate data. NoteLP is a compromise between laborious manual review and automated but opaque LLM results. Rule-based and ML methods of data extraction are complementary. The rule-based approach is a useful option that allows investigators to generate and audit search results to balance efficiency and accuracy.
